# Supplementary material for: The effect of gut passage by waterbirds on the seed coat and pericarp of diaspores lacking “external flesh”: Evidence for widespread adaptation to endozoochory in angiosperms
Source: PLoS One. 2019 Dec 19;14(12):e0226551. doi: 10.1371/journal.pone.0226551 (PMC6922415; doi:10.1371/journal.pone.0226551)
Supplement: S1 Appendix — (DOCX) [file pone.0226551.s001.docx]

**Appendix S1**. **Comparative morphology and anatomy of seed coat/pericarp in control and passed diaspores.** Monocots are presented first followed by eudicots; taxa were arranged alphabetically by their families. Description of each taxon includes: control and passed diaspores as well as and the most likely protection cell layer(s).

***Allium angulosum*** (Alliaceae)

*Control*. Seeds are black, ellipsoid to obovoid, 1.6–2.1 × 0.9–1.4 mm. Surface is reticulate; cells are polygonal with 5–7 sides. Cuticle is 0.5–0.8 mm thick, film-like on the external periclinal walls of exotesta cells, but it forms deposits of granular epicuticular wax resembling a “zipper-like” pattern, 8–12 μm thick, corresponding to the radial cells walls. Seed coat architecture is exotestal, 20–30 μm thick; testa is 3-layerd; exotesta is 11–15 μm thick, taniniferous with external walls lignified, 5–10 μm thick. Innermost layers of testa cells are thin, ± crushed; tegmen is 1–2 cell layers, cellulosic, ± crushed. Figs 1A–B; 3A–D.

*Passed*. 70% of seeds lost their seed coat on 30–90% of their surface; 5% of seeds lost seed coat on < 30% of their surface and 5% seeds preserved seed nearly intact. In the areas where the seed coat was lost, at the surface was left the white-creamy endosperm in which the curved embryo is embedded. Cells walls of endosperm are 4–11 μm thick, cellulosic. Remnants of the innermost tegmen layers may persist on the endosperm surface in some areas. In the areas where the seed coat persisted, the epicuticular wax between epidermal cells was partially stripped (Figs 1C–D; 3E–H).

*Protection layer*: exotesta but even when the entire seed coat is lost, embryos can survive inside the endosperm with thick cell walls.

***Bolboschoenus planiculmis*** (Cyperaceae)

*Control*. Achenes are chestnut-brown, obovate with a short beak, 2.37–2.93 × 2.28–2.61 mm. Surface is smooth when viewed under the stereomicroscope, but it appears indistinctly “honeycombed” under the SEM, with the exocarp cells pentagonal or hexagonal. Silica bodies are absent; cuticle is film-like, 2.4–5 μm thick, unsculptured. Pericarp is 126–261 μm thick; exocarp consists of one layer of radially elongated cells, 60–112 μm thick, with cellulosic external periclinal walls. Upon imbibition or immersion in the water, exocarp cells retain air and likely provide flotation. Mesocarp is a sclerenchyma 60–86 μm thick, consisting of 7–10 layers of longitudinally oriented fibers; the endocarp has one layer of fibers, 18–24 μm thick, transversally oriented. Seed coat is thin and not fused with the pericarp consisting of two cellulosic cell layers (Figs 1E–F; 3I–K).

*Passed*. Color was unchanged; 80% achenes had at least a part of the exocarp removed; 20% of achenes showed cracks or cuticular damage in the exocarp (Figs 1G–H; 3L–M).

*Protection layers*: pericarp mesocarp and endocarp fibers.

***Cyperus flavescens*** (Cyperaceae)

*Control*. Achenes are auburn to reddish brown, obovoid, 0.9–1.2 × 0.5–0.7 mm, apex apiculate; surface is macroscopically smooth but exhibits a lattice of rectangular, longitudinally elongate cells under the SEM. Cuticle is film-like, 0.4–0.8 μm thick. Pericarp is 21.6–33.4 μm thick, with 4–6 (–7) layers of cells. Exocarp cells are flattened with thin, cellulosic cell walls. Mesocarp is reinforced with multiple lignified layers: under the exocarp, one-cell layer with the inner tangential walls lignified and peg-like projections into the cell lumen. Next 2 (–3) cell layers are fibers longitudinally oriented; innermost 1(–2) cell layers (endocarp) are also fibers, but transversally oriented. Tannins are present in all pericarp layers. Seed coat is free from pericarp, with two thin-walled cell layers (Figs 1I, 3N–O).

*Passed*. Color remains unchanged; all achenes have the exocarp removed on 10–30% of their surface; the rest of tissues remain intact (Figs 1J–L; 3Q–R).

*Protection layers*: pericarp mesocarp and endocarp fibers.

***Echinochloa crus-galli*** (Poaceae)

*Control*. Diaspores are spikelets, 2.8–4 × 1.9–3 mm, with two flowers but only the upper flower is fertile and thus one caryopsis is present inside each spikelet. The caryopsis (and seed included in it) are enveloped by the external spikelet structures: the lower glume at the base; upper glume on the dorsal face; sterile lemma on the ventral face; fertile lemma and palea. Total thickness of covering structures is 100–132 μm thick. Glumes and sterile lemma are herbaceous and have complex surface morphologies, which include papillae, various trichomes and silica bodies (they resemble leaf surface). Trichomes can be either bicellular, microscopic (30–60 μm long) or 1-cellular, macroscopic, lignified, and empty inside. Macro hairs are abundant on the veins and increase in frequency and length toward the distal parts of the lemmas and may have role in epizoochory. Anatomically, glumes and sterile lemma/palea consist of a lignified epidermis and 1–3 layers of lignified mesophyll cells, and an inner epidermis with larger cells (especially along the veins), which probably functions as an aerenchyma. Fertile lemma margins cover the fertile palea margins and both are smooth and strongly indurated around the caryopsis at fructification. Fertile lemma and palea are thicker in their median part (80–100 μm thick) and they get gradually thinner towards their margins. Their structure consists of a lignified outer epidermis without trichomes; a mesophyll with a lignified aerenchyma and an internal epidermis with thin cell walls. Pericarp consists of 1(–2) cellulosic layer(s) of cells fused with the seed coat, which is reduced to one single cell layer followed by the aleurone layer of the endosperm (Figs 1L–M; 3S–T).

*Passed*. Passing removed the glumes and sterile lemma; rarely small fragments of these persisted at the base of the diaspores. 90% of caryopses remained enclosed within the fertile lemmas and paleas, which fully preserved their integrity. Superficial abrasions were noted on the fertile lemmas and paleas. In 5% of caryopses, lemma broke longitudinally exposing the caryopsis underneath (Figs 1N–O; 3U–V).

*Protection layers*: tissues of the fertile lemma and palea.

***Cirsium brachycephalum*** (Asteraceae)

*Control*. Cypselae are stramineous yellow, oblong-obovate, sometimes slightly asymmetrical, 2.2–3.4 × 1.1–1.4 mm. Surface of pericarp is smooth; cells are rectangular, elongated; cuticle is film-like, 2–4.2 μm. Pericarp is 98–122 μm thick. Exocarp cells have somewhat thickened cell walls, but cellulosic. Mesocarp is parenchymatic, of 3–4 cells layers; endocarp is 1-layered, with small cylindrical, fiber-like cells. Seed coat is exotestal, at least in part fused with the pericarp. Testa consists of a layer of radially elongated, characteristically lignified cells, 36–48 μm thick and a few parenchymatic, crushed layers (Figs 1Q, 4A–B).

*Passed*. Colour remained unchanged; cypselae uniform; microscopic abrasions and removal of exocarp cuticle were observed on small portions of the achenes (Figs 1R–T; 4C–D).

*Protection layers*: exocarp through its cuticle and seed testa.

***Lychnis coronaria*** (Caryophyllaceae)

*Control.* Seeds reddish-brown (tubercles are dark-gray), reniform to sub-round; 0.9–1.3 × 0.7–0.9 mm. Surface of exotesta is tuberculate; exotesta anticlinal cells walls evenly sinuate, forming numerous acute sinuses. Cuticle is 1.8–3.1 μm thick; epicuticular wax has numerous rodlets connecting through fine threads; sinuses between cells have wax granules. Seed coat is 16–35 μm thick, exotestal. Testa is 19–22.7μm thick, reduced to the exotesta (epidermis) with thick-cell walls impregnated with tannins. Tegmen consists of two layers of cells, thin-walled, and crushed (Figs 2A–B; Figs 4E–H).

*Passed*. All seeds were little affected. Epicuticular waxes were partially stripped from the convex areas of exocarp cells, but persisting in the sinuses. Feces adhered to the sculpture of exotesta (Figs 2C–D; Figs 4I–L).

*Protection layer*: exostesta.

***Cuscuta lupuliformis*** (Convolvulaceae)

*Control*. Seeds are yellow to dark brown, obovoid, 2.5–3.21 × 1.81–2.4 mm. Surface is smooth to finely rugulose; cuticle 0.3–0.6 μm thick; exotesta cells are rectangular; often groups of 2–6 cells are arranged perpendicularly to one another. Seed coat is 98–132 μm thick, endotestal. Exotesta cells contain tanins; cells walls are cellulosic. Mechanical layer is 53–66 μm thick and consists of one lignified palisade layer with linea lucida; only in the hilum area, the palisade layer becomes double. Inner most layers of the seed coat are thin-walled cells, crushed. The embryo is embedded in endosperm (Figs 2E–F; Figs 4M–P).

*Passed*. All the seeds have the epidermis (exotesta) almost entirely removed, leaving exposed the palisade layer. In most seeds, cracks formed in the palisade layer especially around the hilar pad. In 5% of the seeds, the palisade layer cracks extend to the endosperm (Figs 2G–I; 4Q–T).

*Protection layer*: palisade layer of the (meso)testa.

***Elatine hungarica*** (Elatinaceae)

*Control*. Seeds are brown or dark yellow, cylindrical, curved; 560–652 × 204–399 µm, angle of curvature: (246–)273–291(–318)°; surface with rows of rectangular pits [(22–)37–48(–62) pits in the middle row]. Seed coat is 25–35 µm thick, endotestal. Exotesta (epidermis) cells are flattened, thin-walled, cellulosic; cuticle 0.1–0.2 µm. On the concave side of seeds the epidermis forms a “semilunar membrane”. This structure may aid both wind dispersal and flotation. Endotesta is 22–29 µm thick and has strongly lignified, peculiarly thickened cells walls. External cell-walls of the exotesta collapse or rupture revealing the endotesta underneath, which is the tissue that imparts seeds the characteristic pitted aspect. Most of the seeds (80%) undergo this process across the entire epidermis surface (including the semilunar membrane). A reduced number of seeds (20%) preserve their epidermis or this is only partially destroyed. Tegmen has 2 (–3) cell layers, thin-walled and crushed. Figs 2J–K; 5A–B).

*Passed*. Passing did not cause detectable micro-morphological changes in most of the seeds. As an exception, 1–2 seeds had one end broken. Feces often covered the endotestal pits (Figs 2L–M; 5C–E).

*Protection layer*: 1-layer of endotesta cells.

***Elatine hydropiper*** (Elatinaceae)

*Control*. Seeds are similar to those of *E. hungarica* but 498–561 × 190–500 µm, angle of curvation: (220–)265–295(–335)°; surface with rows of pits having the longer side perpendicular to the longitudinal axis of seeds; number of pits in the middle row: (15–)31–42(–59). Seed coat is 22–27 µm thick, with the exotesta, including the semilunar membrane, persistent or partially persistent in most of the seeds. Endotesta is 16–23 µm thick and cells have similar thickenings as *E. hungarica* (Figs 5F–G).

*Passed*. Seeds were virtually unaffected. Passing may collapse some exotesta cells but most remain intact. Feces did not adhere as much as in *E. hungarica* because exotesta is more persistent (Figs 5H–I).

*Protection layer*: 1-layer of endotesta cells.

***Astragalus contortuplicatus*** (Fabaceae)

*Control*. Seeds are goldenrod-yellow with reddish-purple spots, reniform, 0.8–1.1 × 0.5–0.7 mm. 99% seeds did not rehydrate. Surface is smooth macroscopically but appearing reticulate-regulate under the SEM because of the prominent anticlinal cell walls and lamellar epicuticular deposits on the external periclinal walls; cuticle 1.1–1.5 μm thick. Seed coat is 38–42.7μm thick, exotestal. Exotesta consists of radially elongated cells (Malpighian cells), 18–23.8 μm thick, heavily lignified, with a linea lucida; and hourglass cells which are mostly crushed. The tegmen is made of several cell layers ± cellulosic, crushed (Figs 2 N–O; 5J–M).

*Passed*. 76% of seeds changed color to whitish-cream or light brown, which indicates that imbibition took place (and possibly physical dormancy was removed). These seeds had the epicuticular wax partially stripped and fine fissures opened among the distal ends of the Malpighian cells, including in the hilum area; 19% of the seeds were affected only at the level of cuticle; 4% had cracks penetrating through the entire testa; 1% of seeds had fractures of the seed coat reaching to the cotyledons (Figs 2P–Q; 5N–Q).

*Protection layers*: exotesta.

***Glycyrrhiza echinata*** (Fabaceae)

*Control*. Seeds brown to whitish, reniform, unequal in size, 3.21–5.1 × 2.26– 4 mm or 2.11–3.18 × 1.53–2 mm. Surface smooth macroscopically but appearing regulate under the SEM because of epicuticular wax deposits; cuticle 1.8–3.3 μm thick. Seed coat is 82–116 μm thick, exotestal. Exotesta consists of radially elongated cells, 65–76 μm thick, lignified and having a linea lucida (Malpighian cells); hourglass cells are intact. Tegmen consists of several cell layers ± cellulosic, crushed (Figs 2R–S; 5R–S).

*Passed*. Color remained unchanged; 80% of seeds had areas from which the cuticle was partially stripped forming micro-fissures in the distal part of the exotesta; 20% of seeds exhibited deeper cracks which penetrated in the exotesta, but usually did not reach the hourglass cells (Figs 2T–U; 5T–V).

*Protection layer*: exotesta.
