# Supplementary material for: The effect of gut passage by waterbirds on the seed coat and pericarp of diaspores lacking “external flesh”: Evidence for widespread adaptation to endozoochory in angiosperms
Source: PLoS One. 2019 Dec 19;14(12):e0226551. doi: 10.1371/journal.pone.0226551 (PMC6922415; doi:10.1371/journal.pone.0226551)
Supplement: S2 Appendix — (DOCX) [file pone.0226551.s002.docx]

**Appendix S2. Glossary of seed/fruit terms used in the article.**

**Achene –** A simple, dry, indehiscent, one-seeded fruit whose pericarp is mostly free from the seed coat.

**Aleurone layer** **– Layer of cuboid cells found at the periphery of endosperm in Poaceae.**

**Anatropous – An** inverted ovule, bent down and fused with the funiculus; as a result, the micropyle is in the vicinity of funiculus base.

**Berry – A simple, indehiscent fruit with the entire pericarp fleshy.**

**Bitegmic – Ovule with two integuments.**

**Campylotropous – A curved ovule, often kidney-shaped.**

**Capsule** **–** Dry, dehiscent fruit derived two or more fused carpels; containing one or more seeds and opening via pores, longitudinal or transversal sutures.

**Caryopsis** (plural caryopses) **–** A simple, dry, indehiscent, one-seeded fruit in which the pericarp is usually fused with the seed coat (the fruit of Poaceae).

**Cypsela** (plural cypselae) **– Like the** achene, but usually accompanied by a pappus (the fruit of Asteraceae).

**Dehiscent** **– Which opens, with reference to dry fruits such follicles, legumes, capsules, etc.**

**Diaspore – A dispersal “unit” which can be a seed, a fruit, an infructescence or even the entire plant. In this study, only seeds and fruit diaspores were examined.**

**Drupe –** Simple, indehiscent fruit having a fleshy mesocarp and a sclerenchymatous endocarp protecting the seed(s) (usually only one).

**Endocarp – The innermost cell layer(s) of the fruit wall.**

**Endosperm – Trophic tissue of angiosperm seeds which** results through the fertilization of the two polar nuclei in the central cell of the embryo sac by one sperm cell nucleus.

**Endotestal – Seed or type of seed coat architecture in which the mechanical layer(s) develop(s) from the inner epidermis of the outer ovule integument (or single ovule integument in unitegmic ovules).**

**Exocarp – The pericarp epidermis (developed from the ovary epidermis); also called epicarp.**

**Exotestal – Seed or seed coat architecture in which the mechanical layer develops from the outer epidermis of the outer ovule integument (or single integument in the case of unitegmic ovules). This is basically the seed epidermis.**

**Fiber – A much elongated sclerenchyma cell (with thick secondary cell walls, often lignified).**

**Follicle** **– Simple, d**ry, dehiscent fruit, one-locular, one- to many-seeded and derived from a single carpel; dehiscing along a single suture line.

**Funiculus – T**he “cord” through which the ovule is linked to placental tissue.

**Hemi-anatropous –** A type of ovule with the body forming a right angle in relation to the funicle (so it looks like the ovule is lying on its side).

**Indehiscent – which remains closed, with reference to dry fruits such as achenes or caryopses, as well as most of the fleshy fruits.**

**Malpighian cells** **– See** “palisade cells”.

**Mechanical layer – Layer of cells that are most often** lignified, consisting of sclereids, fibers or tracheids; however, thick-cellulosic, silica- or suberin-impregnated cell walls are also possible.

**Mesocarp – Middle layers of the pericarp (located between the exocarp and endocarp).**

**Palisade cells** **– Mechanical layer of sclereids considered by many authors to represent the water barrier that is also responsible for enforcing physical dormancy.**

**Parenchyma – A tissue composed of living cells that may differ in size, shape, and cell wall structure.**

**Pericarp – The fruit wall resulted from the ovary wall. Pericarp consists from exocarp, mesocarp and endocarp.**

**Physical dormancy** **– Seed d**ormancy caused by water-impermeable seed coat or pericarp.

**Physiological dormancy – Seed dormancy has a physiological basis.**

**Seed coat – Totality of seed protective layers developed from the ovule integument(s).**

**Sclereid – A sclerenchyma cell of various shape, but usually not much elongated.**

**Sclerenchyma** **– “Mechanical” tissue composed of cells with thickened secondary cell walls, usually lignified. It includes sclereids and fibers.**

**Tegmen – The portion of the seed coat resulted from the inner ovule integument.**

**Tegmic** **– S**eeds that possess a mechanical layer developed within their former inner integument ovule.

**Testa – The portion of the seed coat resulted from the outer ovule integument. In the case of seeds derived from unitegmic ovules, testa is the entire seed coat.**

**Testal – Seeds that have** their mechanical layer developed from the outer ovule integument (or the only integment when ovules are unitegmic).

**Unitegmic – Ovules with only one integument.**
