# Supplementary material for: The effect of gut passage by waterbirds on the seed coat and pericarp of diaspores lacking “external flesh”: Evidence for widespread adaptation to endozoochory in angiosperms
Source: PLoS One. 2019 Dec 19;14(12):e0226551. doi: 10.1371/journal.pone.0226551 (PMC6922415; doi:10.1371/journal.pone.0226551)
Supplement: S1 Table — (DOCX) [file pone.0226551.s003.docx]

**Table S1. Pearson correlation matrix for diaspore traits.**

| Variable | Marked correlations are significant at p < 0.05000 N=11 (Casewise deletion of missing data) | | | | | | | | | | | | |
| --- | --- | --- | --- | --- | --- | --- | --- | --- | --- | --- | --- | --- | --- |
|  | \| Means \| \| --- \| | \| Std.Dev. \| \| --- \| | \| DiaspLth \| \| --- \| | \| DiaspWth \| \| --- \| | \| CutThick \| \| --- \| | \| A.TotalTh \| \| --- \| | \| B.MechTh \| \| --- \| | \| %Survive \| \| --- \| | \| WetLoad \| \| --- \| | \| WaterPerm \| \| --- \| | \| GermCont \| \| --- \| | \| GermPassed \| \| --- \| | \| Pass-Cont \| \| --- \| |
| \| DiaspLth \| \| --- \| | 1.94718 | 1.20468 | 1.000000 | 0.897963 | 0.442070 | 0.837491 | 0.819461 | -0.193882 | 0.660507 | 0.056001 | -0.175176 | -0.119310 | 0.078315 |
| \| DiaspWth \| \| --- \| | 1.30991 | 0.84971 | 0.897963 | 1.000000 | 0.449808 | 0.838035 | 0.853197 | 0.037255 | 0.756420 | -0.066189 | -0.120333 | -0.035118 | 0.105861 |
| \| CuticleThick \| \| --- \| | 1.52455 | 1.45000 | 0.442070 | 0.449808 | 1.000000 | 0.572554 | 0.376762 | 0.191341 | 0.147293 | 0.374112 | -0.330762 | -0.069447 | 0.321087 |
| \| A.TotalThick \| \| --- \| | 73.17091 | 52.93190 | 0.837491 | 0.838035 | 0.572554 | 1.000000 | 0.936174 | 0.129648 | 0.583503 | 0.053930 | -0.312766 | -0.278755 | 0.066758 |
| \| B.MechThich \| \| --- \| | 44.96091 | 29.97621 | 0.819461 | 0.853197 | 0.376762 | 0.936174 | 1.000000 | 0.051521 | 0.522883 | -0.005209 | -0.140700 | -0.179577 | -0.030190 |
| \| B of A% \| \| --- \| | 66.79455 | 16.40532 | -0.509316 | -0.416745 | -0.593848 | -0.503757 | -0.228917 | -0.193824 | -0.407451 | -0.201485 | 0.396999 | 0.188139 | -0.268912 |
| \| %Survive \| \| --- \| | 18.82364 | 16.60325 | -0.193882 | 0.037255 | 0.191341 | 0.129648 | 0.051521 | 1.000000 | 0.017838 | -0.143291 | -0.251343 | -0.443923 | -0.190798 |
| \| WetLoad \| \| --- \| | 3.55182 | 4.96917 | 0.660507 | 0.756420 | 0.147293 | 0.583503 | 0.522883 | 0.017838 | 1.000000 | -0.527806 | 0.007367 | 0.089676 | 0.090810 |
| \| WaterPerm \| \| --- \| | 1.13900 | 0.06150 | 0.056001 | -0.066189 | 0.374112 | 0.053930 | -0.005209 | -0.143291 | -0.527806 | 1.000000 | -0.465977 | -0.340966 | 0.182093 |
| \| GermCont \| \| --- \| | 0.29818 | 0.26645 | -0.175176 | -0.120333 | -0.330762 | -0.312766 | -0.140700 | -0.251343 | 0.007367 | -0.465977 | 1.000000 | 0.629643 | -0.504246 |
| \| GermPassed \| \| --- \| | 0.30191 | 0.24597 | -0.119310 | -0.035118 | -0.069447 | -0.278755 | -0.179577 | -0.443923 | 0.089676 | -0.340966 | 0.629643 | 1.000000 | 0.353391 |
| \| Pass-Cont \| \| --- \| | 0.00373 | 0.22128 | 0.078315 | 0.105861 | 0.321087 | 0.066758 | -0.030190 | -0.190798 | 0.090810 | 0.182093 | -0.504246 | 0.353391 | 1.000000 |

DiaspLth Diaspore length

DiaspWth Diaspore width

CuticleThick Cuticle thickness

A.TotalThick Total thickness

B.MechThich Thickness of mechanical layers

%Survive Seed survival. Percentage of diaspores recovered intact after gut passage

WetLoad Loading required to break the diaspore when wet

WaterPerm Water permeability (ratio of volume before and after soaking in water)

GermCont Germinability of control seeds

GermPassed Germinability of intact seeds recovered after gut passage

Pass-Cont Difference between the germinability of passed and control seeds.
