# Supplementary material for: The effect of gut passage by waterbirds on the seed coat and pericarp of diaspores lacking “external flesh”: Evidence for widespread adaptation to endozoochory in angiosperms
Source: PLoS One. 2019 Dec 19;14(12):e0226551. doi: 10.1371/journal.pone.0226551 (PMC6922415; doi:10.1371/journal.pone.0226551)
Supplement: S3 Table — (DOCX) [file pone.0226551.s005.docx]

**Table S3. Results of germination tests and effect of gut passage on germinability tested with Fisher’s Exact Test.**

|  | Control | | | Passed | | | Difference in germinability |
| --- | --- | --- | --- | --- | --- | --- | --- |
| Species | Tested | Germinated | Germinability | Tested | Germinated | Germinability | P |
| *Allium angulosum* | 50 | 7 | 0.14 | 129 | 26 | 0.20 | 0.3965 |
| *Astragalus contortuplicatus* | 50 | 16 | 0.32 | 560 | 180 | 0.32 | 1.00 |
| *Bolboschoenus planiculmis* | 50 | 0 | 0.00 | 382 | 10 | 0.03 | 0.6136 |
| *Cirsium brachycephalum* | 50 | 0 | 0.00 | 47 | 4 | 0.09 | 0.0515 |
| *Cuscuta lupuliformis* | 50 | 9 | 0.18 | 156 | 47 | 0.30 | 0.1032 |
| *Cyperus flavescens* | 50 | 0 | 0.00 | 260 | 3 | 0.01 | 1.00 |
| *Echinochloa crus-galli* | 50 | 17 | 0.34 | 76 | 22 | 0.29 | 0.5608 |
| *Elatine hungarica* | 50 | 39 | 0.78 | 82 | 16 | 0.20 | **0.0001** |
| *Elatine hydropiper* | 50 | 18 | 0.36 | 76 | 38 | 0.50 | 0.1445 |
| *Glycyrrhiza echinata* | 50 | 32 | 0.64 | 42 | 23 | 0.55 | 0.3998 |
| *Lychnis coronaria* | 50 | 26 | 0.52 | 52 | 43 | 0.83 | **0.0014** |
