# Supplementary material for: The effect of gut passage by waterbirds on the seed coat and pericarp of diaspores lacking “external flesh”: Evidence for widespread adaptation to endozoochory in angiosperms
Source: PLoS One. 2019 Dec 19;14(12):e0226551. doi: 10.1371/journal.pone.0226551 (PMC6922415; doi:10.1371/journal.pone.0226551)
Supplement: Attachment — Submitted filename: Response to reviewers.docx [file pone.0226551.s006.docx]

Dear Editor,

We have addressed the request of the editorial office and carefully considered/implemented the suggestions of the reviewers. Please see below the rebuttal to each point raised by the reviewers.

***Editor***

*1. In your Methods section, please provide additional information regarding the permits you obtained for the work. Please ensure you have included the full name of the authority that approved the field site access and, if no permits were required, a brief statement explaining why."*

Authors: Added the following text (lines 147-148): “No field site access permit was required because seeds were harvested from unprotected areas”.

***Reviewer #1****: The paper is interesting and is suitable for publication. One major question is arisen: are you really sure that the "Ex" in Cirsium is really seed coat. We have studied some Asteraceae (published or in review) and recognized that the sclerenchyma belongs to the pericarp, and the seed coat is represented by two or several parenchymatous cell layers. Based on that, I ask you to check whether the exotestal layer of the seed coat is really a part of seed coat and not a part of pericarp.*

Authors: We have verified again the identity of the mechanical layer in *Cirsium* (Carduoideae, Asteraceae) and confirm that this cell layer is indeed the seed coat exotesta, and that it does not belong to the pericarp. The reviewer, Dr. Alexander Sukhorukov, studied *Tragopogon* and more generally *Scorzonerinae* in Asteraceae, which have a different organization of the seed coat/pericarp architecture. In these latter groups, the defense layers has indeed been transferred from the seed coat to the pericarp. The following references, among others, confirm our decision:

Häffner, E., 2000. On the phylogeny of the subtribe *Carduinae* (tribe Cardueae, Compositae). Englera, pp.: 3-208.

Ozcan, M., 2017. Cypsela micromorphology and anatomy in *Cirsium* sect. *Epitrachys* (Asteraceae, Carduoideae) and its taxonomic implications. Nordic Journal of Botany, 35: 653-668.

Ozcan M. 2019. Carpological investigations on some *Cirsium* (Asteraceae, Carduoideae) taxa from NE Anatolia. Acta Botanica Hungarica. 61: 369-385.

Roth, I. 1977. Fruits of Angiosperms; Gebruder Borntraeger, Berlin, Stuttgart; pp.: 258-270.

Zarembo, E.V. and Boyko, E.V. 2008. Carpology of some East Asian *Cardueae* (Asteraceae). Anales del Jardín Botánico de Madrid. 65: 129-134.

***Reviewer #2****: The experiment was well designed and the data collected and analyzed with sound technical methods. The data supported the conclusions and summaries. A well written paper!

Minor revision suggested in:
1. Have the controlled seeds going through the same preparation treatment? i.e., disinfection treatment ( 1 minutes in 25% NaOCL). This not clearly stated in the section "2. Germination tests".*

Authors: we made an addition (line 172) to indicate that both control and treated diaspores were treated the same way.

*2. Provide a brief justification for the same germination conditions (i.e., light period and temperature alternation) for all 11 species, in the section "2. Germination tests".*

Authors: That would not be necessary because as indicated in the text, the objective was to determine if the seeds can germinate, not to determine their specific germination requirements.

Sincerely,

Mihai Costea

Department of Biology;

Wilfrid Laurier University;

75 University Avenue West,

Waterloo, ON, N2L 3C5, Canada

Phone: 519 884-0710; 3407 (office) or 2887 (lab)
